# Rhino-Orbital Mucormycosis in a COVID-19 Patient Co-Infected With Klebsiella Pneumoniae

**DOI:** 10.7759/cureus.17249

**Published:** 2021-08-17

**Authors:** Lovish Wadhwa, Shikhar Khurana

**Affiliations:** 1 Internal Medicine, Ramaiah Medical College, Bangalore, IND; 2 Internal Medicine-Neurology, Ramaiah Medical College, Bangalore, IND

**Keywords:** rhino-orbital mucormycosis, covid-19, klebsiella pneumonia, diabetes mellitus, posaconazole, otorhinolaryngology, ophthalmology

## Abstract

We present a case of a 34-year-old male with coronavirus disease 2019 (COVID-19) and uncontrolled blood sugar with right orbital cellulitis and right ethmoidal and maxillary sinusitis. Histopathology of a middle meatus tissue biopsy collected via diagnostic nasal endoscopy (DNE) revealed the presence of *Mucor* and *Klebsiella pneumoniae (K. pneumoniae).* After the administration of intravenous (IV) insulin, cefoperazone-sulbactam, and posaconazole (due to the non-availability of amphotericin B), he underwent surgical debridement and functional endoscopic sinus surgery (FESS). After being on antibiotics and antifungals for two weeks post-surgery, the patient had a favorable outcome and further tests revealed improved blood counts and inflammatory markers.

## Introduction

The outbreak of severe acute respiratory syndrome coronavirus 2 (SARS-CoV-2) has turned into a global healthcare crisis, with the pandemic hitting India severely in 2021. Secondary infections are a well-defined phenomenon associated with viral infections like influenza, SARS [coronavirus disease 2019 (COVID-19)], the Middle East respiratory syndrome (MERS)-related coronavirus, etc. One such secondary infection that has become an epidemic in India is mucormycosis [1]. A rare opportunistic fungal infection caused by molds of the order Mucorales, mucormycosis is a highly invasive and ruthlessly progressive disease [2]. These fungi primarily affect patients with diabetes (type 2 more frequently than type 1) or a compromised immune function. While the fungus shows a proclivity for the rhino-orbital tract, it infrequently presents as a pulmonary, cutaneous, or gastrointestinal form. In this report, we discuss a rare case of a COVID-19 patient secondarily infected with invasive rhino-orbital mucormycosis along with a *Klebsiella pneumoniae (K. pneumoniae)* co-infection.

## Case presentation

A 34-year-old male patient initially presented to the outpatient department with a history of fever for 15 days, which had been followed by moderate swelling, redness, and irritation in the right eye along with a right-sided headache for one week. He was advised to get a contrast-enhanced CT (CECT) of the head and orbit; diagnostic nasal endoscopy (DNE) was deferred until the patient was tested for SARS-CoV-2 in view of the ongoing COVID-19 pandemic. He was started on antibiotics and was advised home isolation till the reports were out.

**Figure 1:**
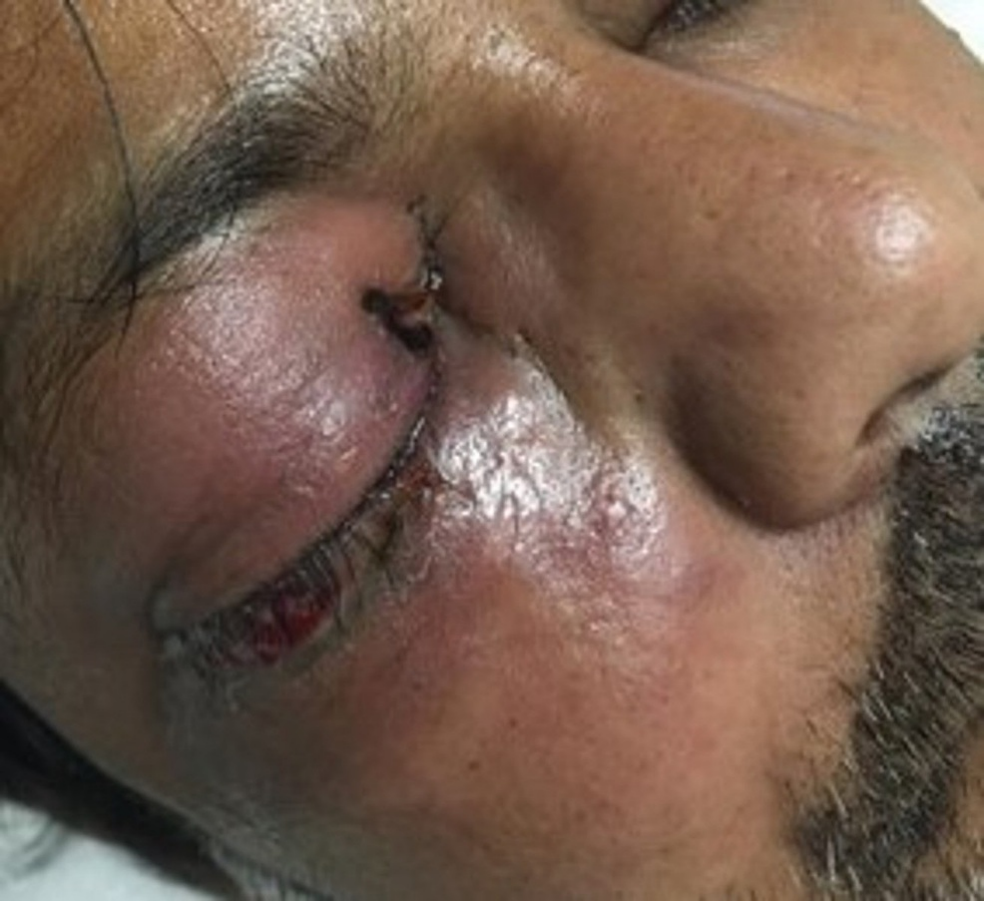
Right periorbital erythema and swelling with black eschar near the medial canthus

The patient returned to the hospital emergency department (ED) two days later, with complaints of increased right eye pain and redness along with reports. He was admitted the same day for further management and evaluation. Upon admission (day 0), the patient was hemodynamically stable, conscious, alert, and oriented, but had a high-grade fever. Physical examination revealed visible proptosis and conjunctival congestion in the right eye with erythema and swelling in the periorbital area on the same side. Cutaneous involvement included erythema over the right side of the nose and maxillary area. A small black eschar was noted near the medial canthus of the right eye over the upper eyelid, as shown in Figure 1. Extraocular movements of the right eye were painful.

On detailed history taking, the patient gave no history of diabetes mellitus, hypertension, or any other chronic illnesses, and no history of similar complaints in the past with respect to self or any family members. The patient mentioned that he had taken some medications he had bought from a local pharmacy, but could not name them (we assumed them to be dexamethasone). The patient had used to consume six standard drinks of alcohol every day for 12 years, which he had quit three years back. He had also used to consume oral tobacco for 14 years, which he had stopped one year back.

The patient registered a positive SARS-CoV-2 reverse transcription-polymerase chain reaction (RT-PCR). Respiratory examination revealed the presence of bilateral crepitations on auscultation of lung fields. His chest X-ray was normal, but he had bilateral multifocal ground-glass opacities detected on high-resolution CT (HRCT) thorax with a CT severity score of 4/25. Cardiovascular and abdominal examinations were unremarkable.

The CECT of the head and orbit showed thickening of the pre-septal and the adjacent subcutaneous fat of the face and nose with inflammatory fat stranding in the retro-orbital fat, indicating right orbital cellulitis. Proptosis of the right eyeball due to increased extraocular muscle bulk was also noted. Soft-tissue densities were seen in the right ethmoidal and maxillary sinuses, consistent with sinusitis, with inflammation extending to the right orbit via gaps of lamina papyracea. Upon admission, the patient’s lab studies showed an HbA1c of 12.7% and a random plasma glucose value of 551 mg/dl without the presence of acidosis in arterial blood and absence of ketonuria, though the patient had never been diagnosed with diabetes mellitus, possibly owing to lack of health checkups due to his low socioeconomic background. The complete blood count (CBC) showed an acute rise in total leukocyte count at 21,500/uL (with a neutrophil-lymphocyte ratio of 14.5). The patient also had deranged inflammatory markers, with an acute increase in alkaline phosphatase (ALP), C-reactive protein (CRP), lactate dehydrogenase (LDH), procalcitonin, d-dimers, and ferritin.

Promptly on day one, the patient was given 10 units of subcutaneous regular human insulin and then maintained on a 70/30 mixture of intermediate-acting and regular insulin. He was also started on broad-spectrum antibiotics for invasive cellulitis and sinusitis with a third-generation cephalosporin combined with a beta-lactamase inhibitor (IV cefoperazone-sulbactam) and IV teicoplanin. As per the hospital’s COVID protocols, the patient was started on antitussives, vitamin C, zinc, anti-coagulant (low-molecular-weight heparin) due to very high d-dimers, as well as remdesivir with favipiravir.

On day two, the patient complained of increased swelling and pain in the right eye and right side of the nose. He also started experiencing paresthesia over the right periorbital area, right side of the nose, right maxillary area, and right palate inside the oral cavity. On day three, a purulent discharge was identified to be arising from the swollen eye, associated with a decrease in visual acuity and a blood-stained purulent discharge from the right nostril. As day three progressed, intra-ocular secretions increased, and he could not open his right eye. The nasal discharge and right eye wound swab specimens were sent for microbiological culture but returned negative for any pathogen after 24 and 48 hours.

Otorhinolaryngology and Ophthalmology reviews were sought, and the patient was advised to undergo an MRI; he was also scheduled for a DNE, due to suspicion of a rhino-orbital-sinusoidal mucormycosis. In light of the aforementioned observations and the non-resolution of symptoms, the patient was also started on a potent triazole antifungal, oral posaconazole 800 gm/day in two divided doses (due to the non-availability of IV amphotericin B). He was additionally started on moxifloxacin and amphotericin B eye drops by the ophthalmologist.

**Figure 2:**
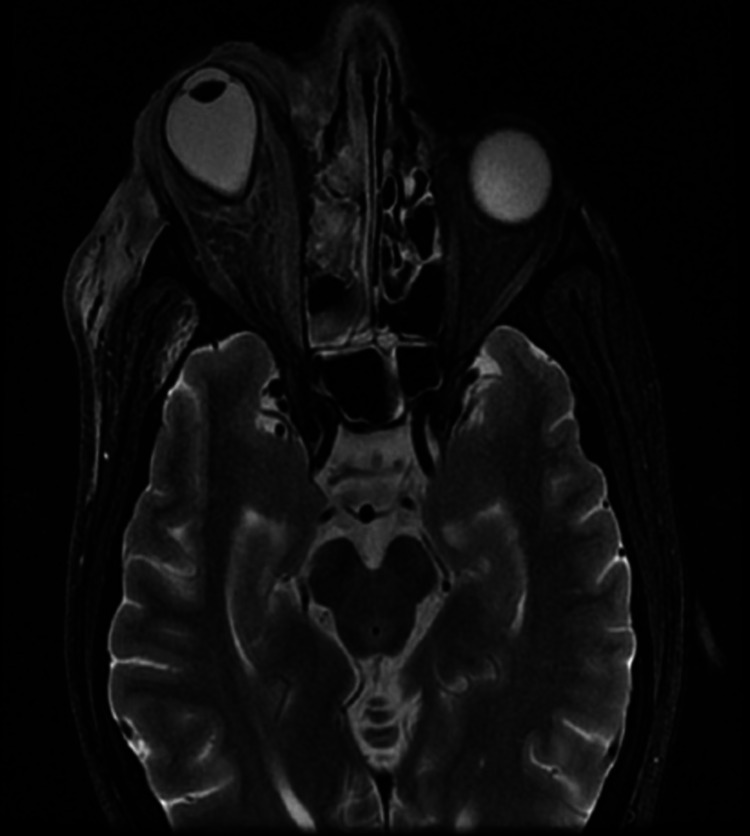
MRI T2 brain axial view The image shows right orbital cellulitis and sinusitis (indicated by proptosis, enhancement of soft tissue, and sinuses) MRI: magnetic resonance imaging

**Figure 3:**
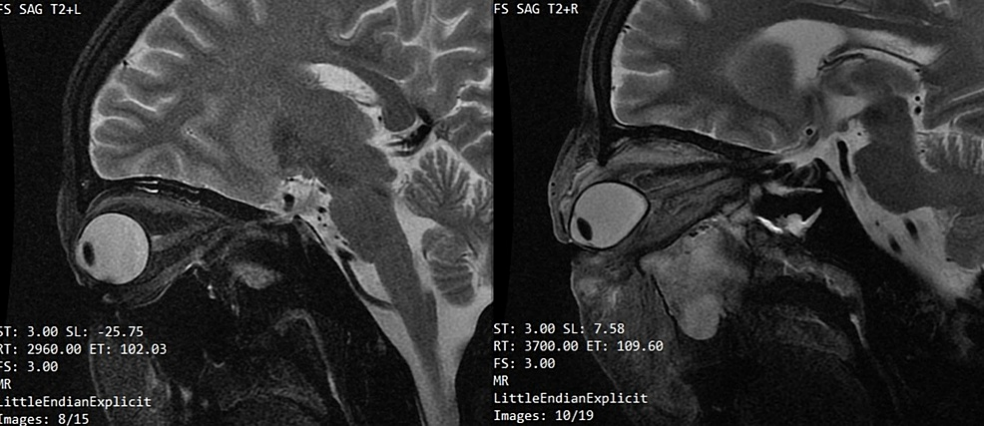
MRI T2 sagittal view (left and right orbital) The images show right eye proptosis, extraocular muscle hypertrophy, and enhancement of periorbital soft tissue MRI: magnetic resonance imaging

**Figure 4:**
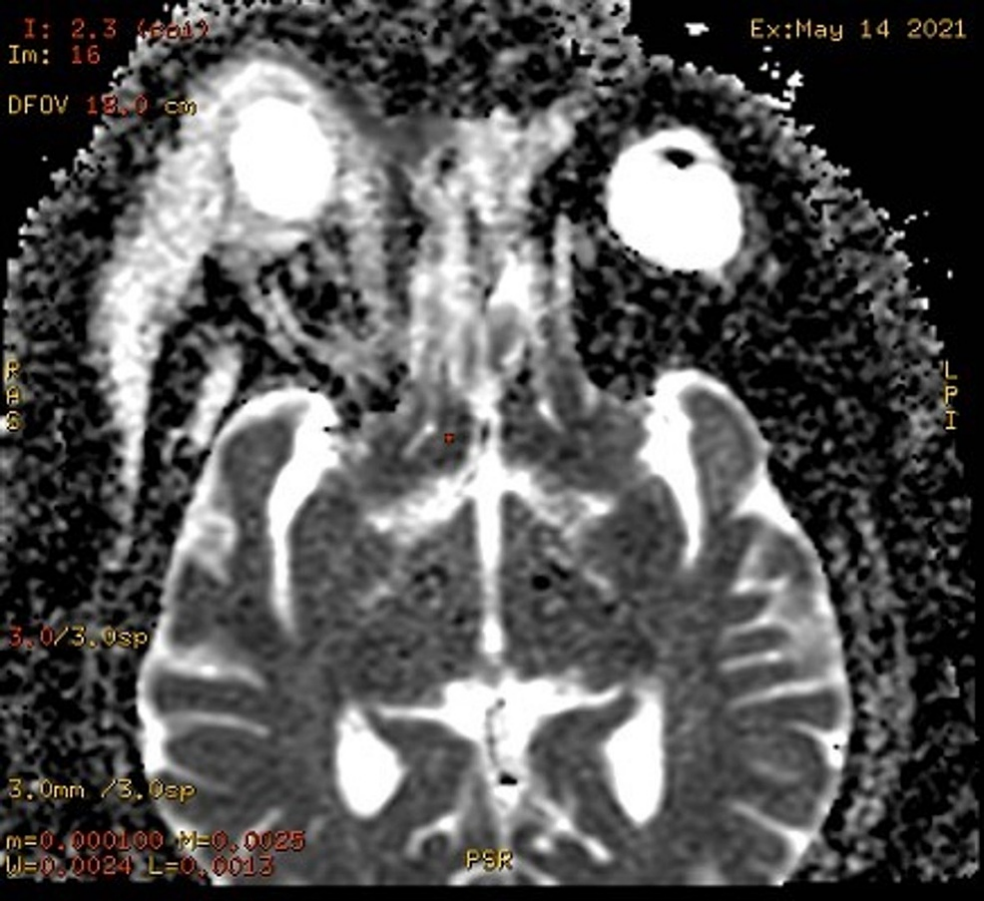
MRI DWI brain - sagittal view with contrast The image shows heterogeneous enhancement in the pre-septal orbit around the sclera with mild mucosal enhancement in the right maxillary and ethmoidal sinuses MRI: magnetic resonance imaging; DWI: diffusion-weighted imaging

On day four, the patient underwent an MRI brain, paranasal sinus, and orbit with contrast, which showed evidence of proptosis of the right eye with an altered globe shape and a conical bulge at the optic nerve head with edema and inflammatory changes in pre-septal orbit. There was also enlargement of extraocular muscles (inferior and medial rectus) with complete blockage of the nasolacrimal duct. Complete opacification and rarefaction of the superior medial wall of the right maxillary sinus along with partial opacification of ethmoidal air cells were also observed. Post-contrast images showed ill-defined heterogeneous enhancement in the pre-septal orbit around the sclera with mild mucosal enhancement in the right maxillary and ethmoidal sinuses. This confirmed the findings of infective orbital cellulitis and myositis secondary to dacryocystitis but limited to the pre-septal region of the right orbit as shown in Figures 2-4. There was no notable intracranial or dural venous sinus extension with evidence of normal brain parenchyma.

**Figure 5:**
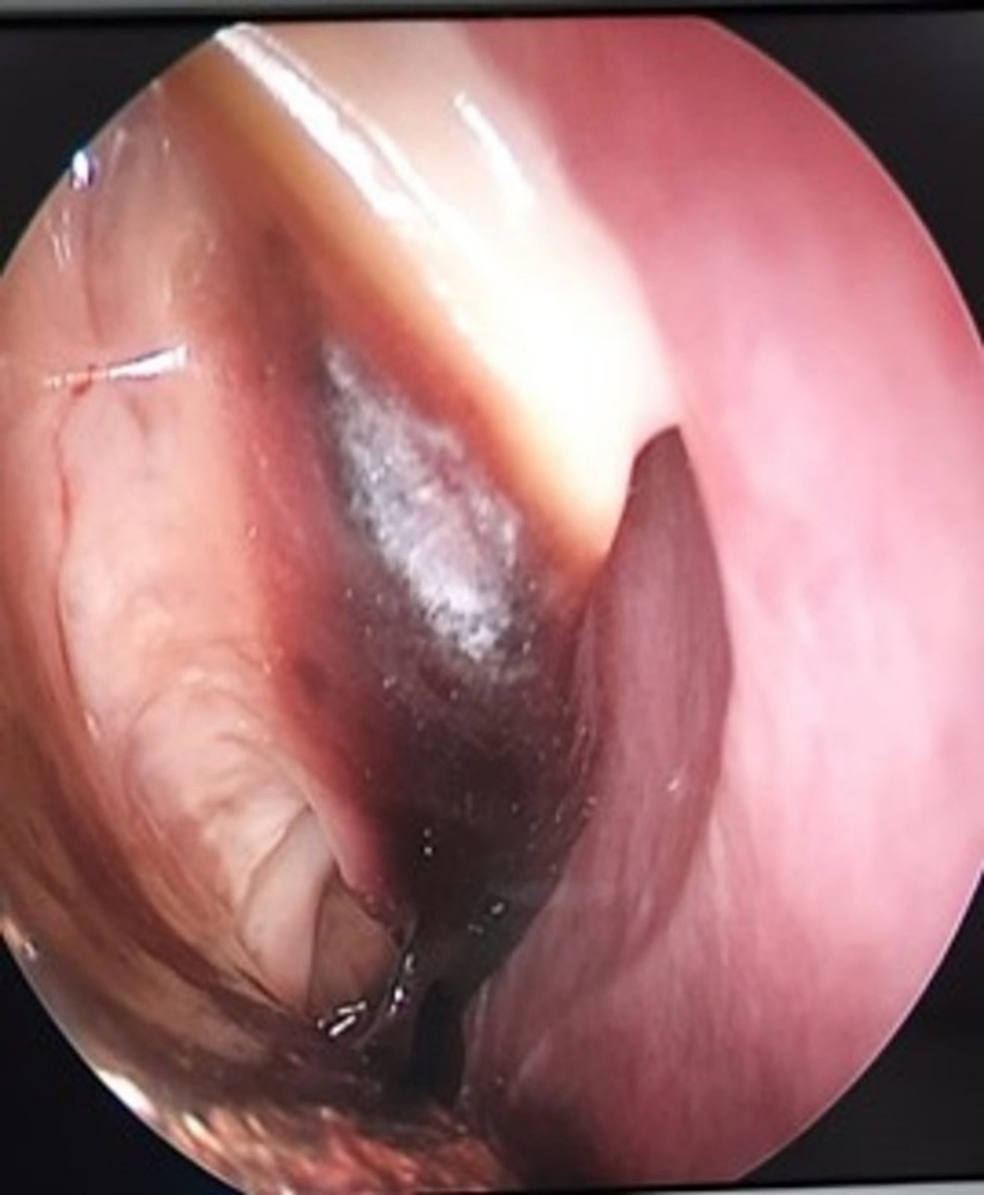
DNE showing necrotic debris in the middle meatus, axilla, and roof of the right nasal cavity DNE: diagnostic nasal endoscopy

**Figure 6:**
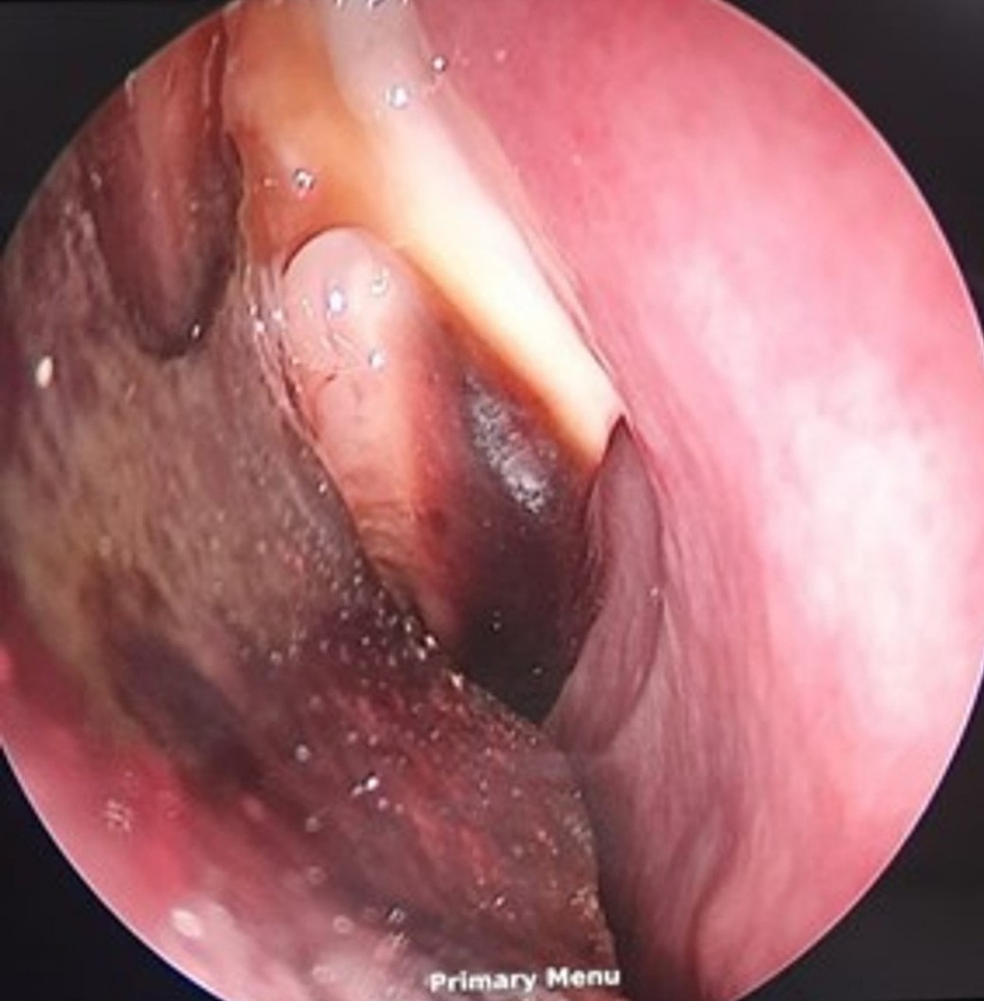
DNE images showing blackish discoloration over middle and inferior turbinates and lateral wall DNE: diagnostic nasal endoscopy

The DNE showed blackish discoloration of the middle and inferior turbinate and the right lateral wall. Endoscopy also showed necrotic debris in the middle meatus, axilla, and roof of the right nasal cavity as shown in Figures 5, 6.

**Figure 7:**
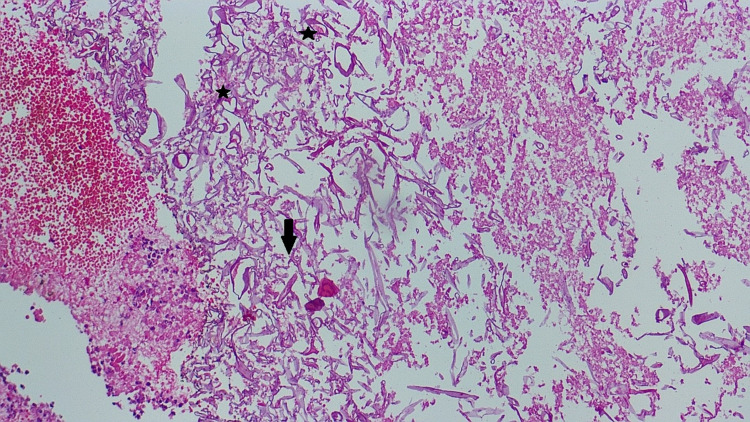
Histopathological slide of middle meatus tissue biopsy The image shows branching septate at right angles (arrow) and multiple fungal sporangia (star) - highly suggestive of mucormycosis

A 5 x 3-mm-size middle turbinate biopsy was taken during the procedure and sent for culture and histopathology, which revealed broad aseptate hyaline ribbon-like hyphae branching at 90 degrees amid necrotic debris and neutrophilic nuclei, most likely suggesting mucormycosis (Figure 7).

The tissue was also sent for a 48-hour aerobic culture and sensitivity and returned positive for wild-type *K. pneumoniae*, sensitive to cefoperazone-sulbactam but resistant to most other common antimicrobials.

On day six, the patient underwent surgical debridement of necrotic tissue and functional endoscopic sinus surgery (FESS), which included modified endoscopic Denker’s approach for endonasal maxillotomy, orbital decompression by removing ethmoidal air cells and lamina papyracea, frontal sinusotomy, and sphenoidectomy. Postoperatively, the patient was continued on oral posaconazole for two more weeks. The labs showed a steady decline in total leucocyte count at 7,000/uL on postop day 10, and the inflammatory markers also returned to normal levels.

## Discussion

Mucormycosis is a life-threatening fungal infection caused by ubiquitous, saprophytic opportunistic fungi of the order Mucorales belonging to seven families, all of which can cause mucormycosis. An upsurge of mucormycosis has been reported throughout the world over the past two decades; however, its rise in developing countries including India has been phenomenal. This increasingly high incidence of mucormycosis in India has been attributed primarily to a continued increase in the patient population with uncontrolled diabetes, which is one of the major risk factors for this disease in developing countries [3]. With the COVID-19 pandemic hitting the country harshly, along with the prevalent and unsupervised use of glucocorticoids in the treatment of early COVID-19, mucormycosis cases have sky-rocketed. Many states within the country have declared the disease an epidemic [4]. The steroid treatment is known to precipitate type 1 or type 2 diabetes in patients who are already borderline diabetics or are in the process of developing diabetes.

Besides hyperglycemia, predisposing factors for mucormycosis include solid organ or hematopoietic stem cell transplantation (HSCT), malignancy, neutropenia, or glucocorticoid treatment. Elevated levels of free iron have also been a common finding, which is proposed to support fungal growth in serum and tissues [5]. Additionally, at times, mucormycosis may present in a previously undiagnosed diabetic patient with the first clinical recognition of hyperglycemia, as was the case with our patient, often masked by glucocorticoid use. Patients with specific defects in host defense tend to have a predilection for one form of the disease; for instance, patients presenting with diabetic ketoacidosis (DKA) typically develop the rhino-orbital-cerebral form and much more rarely develop pulmonary or disseminated disease [6]. Mucormycosis infections are characterized by extensive angioinvasion that results in vessel thrombosis and subsequent tissue necrosis [5,7,8]. This angioinvasion contributes to the capacity of the organism for hematogenous dissemination to other target organs. Consequently, damage of and penetration through endothelial cells or the extracellular matrix proteins lining blood vessels is a critical step in the pathogenesis.

Mucormycosis has a broad range of clinical manifestations. The nomenclature of the mycosis is suggested by anatomical site localization and clinical presentation. For the head and neck region, they are usually classified into isolated nasal, rhino-orbital, or rhino-orbital-cerebral. Other recognized forms include pulmonary, cutaneous, gastrointestinal, disseminated, and miscellaneous [2,6]. Rhino-orbital-cerebral mucormycosis (ROCM) happens to be the most common. If left untreated, infection usually spreads from localized nasal infection to the orbit through the ethmoid sinus, resulting in the compromise of extraocular muscle function, proptosis, chemosis, and ultimately irreversible blindness. From the orbit, spread often takes place via hematogenous or contiguous dissemination to the frontal lobe of the brain, causing lethargy, seizures, abscesses, and thrombosis via venous drainage to the cavernous sinus, characterized by bilateral orbital involvement and ophthalmoplegia. Pertaining to our case, the initial symptoms of sinusitis and periorbital cellulitis indicated a rhino-orbital mucormycosis, including intra-orbital ache and right-sided pain and numbness in the areas supplied by the ophthalmic and maxillary branches of the trigeminal nerve (CN V1, V2). A black necrotic eschar was also observed near the medial canthus of the right eye in our patient, which has been defined as a hallmark of mucormycosis, thus raising our suspicion towards mucormycosis [5]. However, the absence of this finding should not exclude the possibility of mucormycosis.

A high index of suspicion is required for the diagnosis of mucormycosis. Definitive diagnosis requires a positive culture from the infected site (an aspirate or tissue biopsy specimen) or histopathologic evidence of mucormycosis. Biopsy with histopathologic examination remains the most sensitive and specific modality for definitive diagnosis [9]. Biopsy reveals characteristic wide (≥6 to 30-μm), thick-walled, ribbon-like, aseptate hyphal elements that branch at right angles. Imaging techniques such as CT scanning and MRI are used to determine the exact location and extent of infection. MRI is more sensitive (80%) for detecting orbital and CNS disease than CT [10]. It has additionally been recommended that high-risk patients undergo a diagnostic endoscopy and/or surgical exploration as the early establishment of the diagnosis and prompt surgical intervention aids in controlling the extent and severity of the disease. The biopsy specimen was cultured for bacteria, and interestingly, revealed the growth of *K. pneumoniae* that was resistant to ceftriaxone, cefuroxime, ciprofloxacin, and tigecycline. A 2020 study [11] showed that there has been an increasing trend in the isolation rate as well as the resistance of *K. pneumoniae* to most of the regularly prescribed antimicrobials. Sensitivity of the *K. pneumoniae* in our patient was predominantly shown for cefoperazone-sulbactam therapy, which was coincidentally the antibiotic started by us initially.

To the best of our knowledge, there has been only one reported case of co-infection with mucormycosis and *K. pneumoniae *so far, but in contrast to our patient, that was a case of isolated cutaneous mucormycosis [12].

Mucormycosis treatment guidelines emphasize on early initiation of therapy as waiting for cultures may lead to delay and further deterioration of the symptoms. Rapid reversal of underlying predisposing risk factors is also strongly advised, such as treating hyperglycemic patients with fluids and insulin. Hence, patients should be empirically treated for mucormycosis whenever risk factors for infection are present and positive cultures and/or compatible clinical syndromes are suspected [8,9].

Liposomal amphotericin B (LAmB), a polyene antifungal agent, administered intravenously at a dose of 5-10 mg/kg once a day, is the drug of choice. It is preferred over conventional amphotericin B deoxycholate since it is less nephrotoxic, has better CNS penetration, and has shown better outcomes in a retrospective clinical review, but is more expensive [9]. Due to the pandemic, we encountered an acute dearth of pharmacological resources, causing liposomal amphotericin B to be unavailable in our hospital. Posaconazole, a broad-spectrum triazole antifungal, was used to treat our patient. Posaconazole is usually employed as a step-down or salvage therapy for patients who have responded to amphotericin B or those who cannot tolerate it. Posaconazole is much more active than other azoles against many Mucorales species and has been successfully used as a sole antifungal to treat invasive mucormycosis [13,14]. Aggressive surgical debridement of involved tissues should be considered as soon as the diagnosis of any form of mucormycosis is suspected. Surgical intervention with removal of necrotic tissue and debulking infection has been associated with improved survival in anecdotal clinical reviews of rhino-cerebral and pulmonary infection [9,15]. More recent evidence shows that endoscopic debridement with limited tissue removal can be accomplished to avoid potential gross disfigurement. This was carried out in our patient under the bracket surgery FESS [16], encompassing opening up of the maxillary sinus and removal of the necrosed ethmoidal air cells and sphenoid cavity. The patient showed good recovery with cefoperazone and posaconazole, proving their effectiveness in co-infected cases such as this.

## Conclusions

There are still concerns about the cause and increased incidence of mucormycosis in COVID-19 patients as a secondary infection/co-infection or a post-COVID-19 implication. Though mucormycosis is highly prevalent in diabetics, the unrestricted, imprudent, and, in many cases, self-prescribed use of glucocorticoids and immunosuppressants for treating SARS-CoV-2 may have a higher likelihood of contributing to this fungal epidemic. Co-infection with other bacterial or fungal agents can lead to resistant symptoms if not properly investigated. Further research is necessary for better prevention and management of opportunistic infections in COVID-19 patients. Furthermore, caution should be exercised in the use of immunosuppressants, along with continuous monitoring.
